# Exploiting Quorum Sensing Interfering Strategies in Gram-Negative Bacteria for the Enhancement of Environmental Applications

**DOI:** 10.3389/fmicb.2015.01535

**Published:** 2016-01-08

**Authors:** Weiwei Zhang, Chenghua Li

**Affiliations:** Department of Aquaculture, School of Marine Sciences, Ningbo UniversityNingbo, China

**Keywords:** quorum sensing, autoinducer, QS regulation, wastewater treatment system, aquaculture

## Abstract

Quorum sensing (QS) is a widespread intercellular form of communication to coordinate physiological processes and cooperative activities of bacteria at the population level, and it depends on the production, secretion, and detection of small diffusible autoinducers, such as acyl-homoserine lactones (AHLs), auto-inducing oligo-peptides (AIPs) and autoinducer 2. In this review, the function of QS autoinducers of gram-negative bacteria in different aspects of wastewater treatment systems is examined. Based on research primarily performed over the past 10 years, QS involvement in the formation of biofilm and aerobic granules and changes of the microbial community and degradation/transformation pathways is discussed. In particular, the QS pathway in the role of bacterial infections and disease prevention in aquaculture is addressed. Interference of QS autoinducer-regulated pathways is considered potential treatment for a variety of environmentally related problems. This review is expected to serve as a stepping stone for further study and development strategies based on the mediation of QS-regulated pathways to enhance applications in both wastewater treatment systems and aquaculture.

## Introduction

Cell-cell communication is ubiquitously used in microbes and microbial communities to monitor and adapt to their external environment via contact-based chemical exchanges (Lobedanz and Søgaard-Andersen, 2003; Phelan et al., 2012), chemical signaling (Eberhard et al., 1981; Hussain et al., 2008; Schaefer et al., 2008; Galloway et al., 2011) and electric signaling (Nielsen et al., 2010; Shrestha et al., 2013). In one such system, quorum sensing (QS), bacteria can “count” their local population numbers using autoinducers, which are small chemical molecules. QS was first discovered in the marine bacterium *Vibrio fischeri* (Nealson et al., 1970) and later was coined by Fuqua et al. (1994), referring to the acylated homoserine lactone (AHL)-mediated luxR/luxI regulated system.

**Figure 1:**
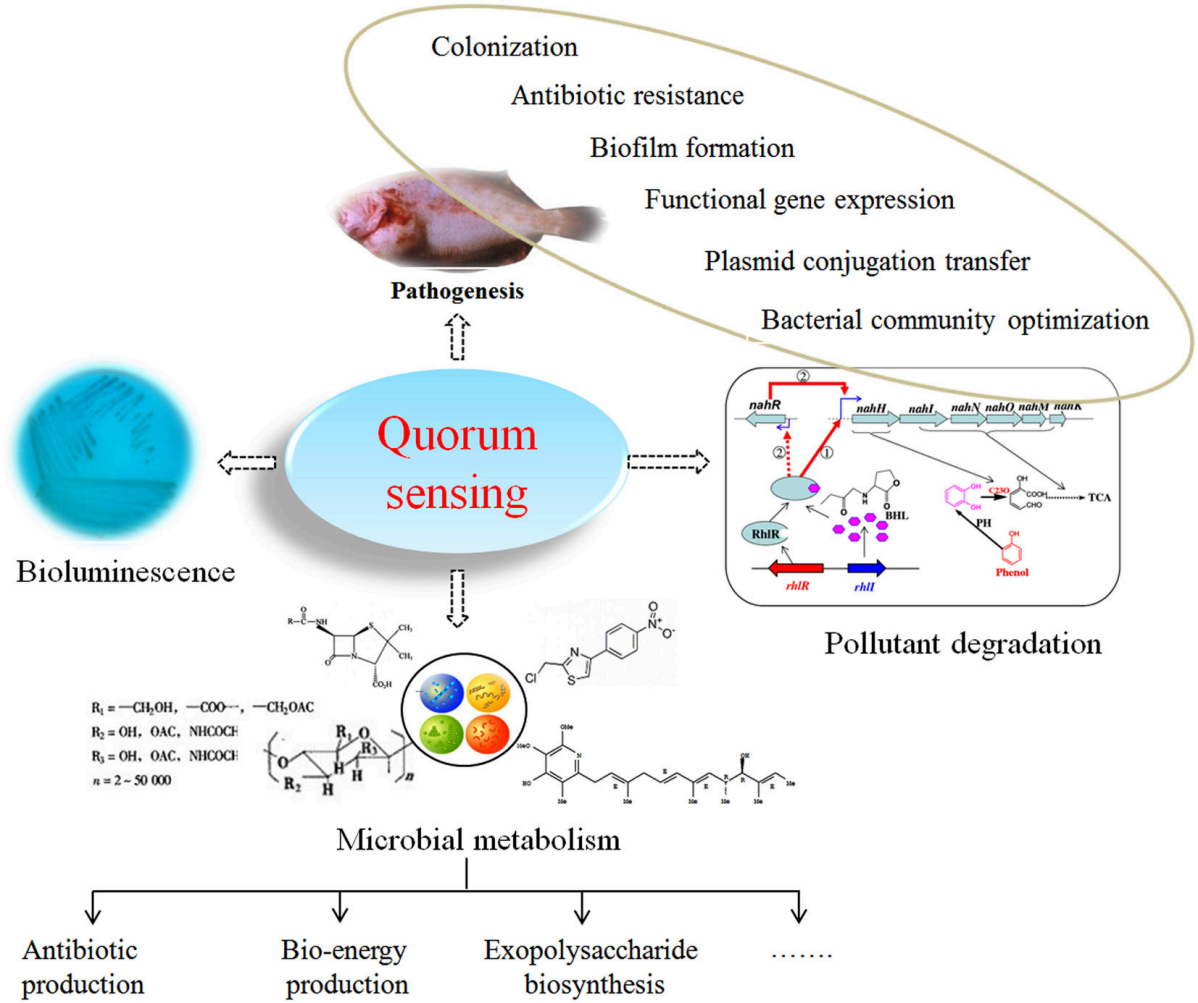
**The various phenotypes regulated by autoinducer-induced QS systems**.

In QS bacteria, when the concentration of autoinducer accumulates to a certain threshold, autoinducers bind to transcriptional regulators to alter gene expression profiles (Miller and Bassler, 2001; Di Cagno et al., 2011; Schaefer et al., 2013) or bind to extracellular domains of specific membrane histidine kinase receptors followed by autophosphorylation and a cognate cytoplasmic response regulator (Sturme et al., 2002; Taga et al., 2003; Neiditch et al., 2006; Thoendel et al., 2011; Ke et al., 2015). Autoinducers regulate the expression of QS-dependent genes on a population-wide scale, endowing bacteria with the ability to live in a “society” that controls many important physiological processes and to initiate “co-operative” behaviors, such as biofilm development (Sakuragi and Kolter, 2007; Shepherd and Lindow, 2009; Liu et al., 2011; Anbazhagan et al., 2012; Ren et al., 2013), pathogenesis (Smith et al., 2007; Natrah et al., 2012; Decker et al., 2013; Pande et al., 2013), and pollutant biodegradation (Toyofuku et al., 2007; Yong and Zhong, 2010, 2013b; Wang et al., 2013a,b; Yong et al., 2015; Figure 1). These activities are unlikely to be completed when only one individual bacterium undertakes the task; therefore, QS has a significant impact on the environment (Jayaraman and Wood, 2008; Galloway et al., 2012), healthcare (Biradar and Devi, 2011; Jamuna Bai and Ravishankar Rai, 2011; Galloway et al., 2012; Yong and Zhong, 2013a) and agriculture (Kalia, 2013). Recently, a number of excellent reviews have highlighted the roles of QS systems, particularly the disruption of QS systems, in water treatment systems (Nguyen et al., 2012; Feng et al., 2013; Lade et al., 2014; Siddiqui et al., 2015; Yong et al., 2015), biodegradation (Díaz et al., 2013; Yong et al., 2015), and pathogenesis control (Bhardwaj et al., 2013; LaSarre and Federle, 2013) as well as in the development of novel disease diagnosis strategies and antimicrobial agents (Roy et al., 2011; Nafee et al., 2014; Rampioni et al., 2014). In this review, a selection of recent case studies demonstrating progress in the development of autoinducer-mediated QS systems used by gram-negative bacteria is summarized, and a systematic review of the manipulation of autoinducer-mediated QS systems in wastewater treatment systems and aquaculture is presented.

## Basics of QS signaling molecules

**Figure 2:**
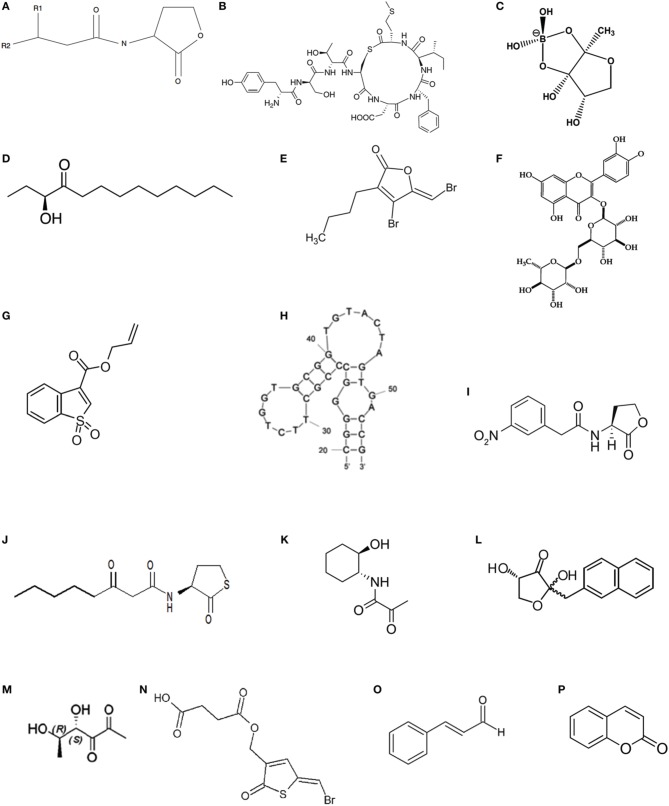
**Chemical structures of specific QS signaling molecules, QSIs and agonistic analogs of QS signaling molecules**. **(A)** Basic structure of the AHL signaling molecule where R1 = H, OH, or O and R2 = C1–C18; **(B)** AIP-1; **(C)** AI-2; **(D)** CAI-1 C13-AHK; **(E)** (5Z)-4-bromo-5-(bromomethykene) -3-butyl-2(5H)-furanone; **(F)** rutin; **(G)** allyl benzo[b]thiophene-3-carboxylate 1,1-dioxide; **(H)** homoserine lactone (HSL) aptamer; **(I)** N-(3-nitro-phenylacetanoyl)-L-homoserine lactone; **(J)** N-(3-oxo-acyl)-homocysteine thiolactones; **(K)** N-(3-oxo-acyl)-trans-2-aminocyclohexanols; **(L)** naphthyl-DPD; **(M)** (4S,5R)- -dihydroxyhexanediones; **(N)** (Z)-4-((5-(bromomethylene)-2-oxo- 2,5-dihydrothiophen-3-yl)-4-oxobutanoic acid); **(O)** cinnamaldehyde; **(P)** coumarin.

Three types of signaling molecules are generally produced and secreted by QS bacteria (Di Cagno et al., 2011). Acyl-homoserine lactones (AHLs, Figure 2A) are the best known class of autoinducers and are frequently used by gram-negative bacteria (Galloway et al., 2011), whereas autoinducing peptides (AIPs, Figure 2B) are the major autoinducers produced by gram-positive bacteria (Sturme et al., 2002; Thoendel et al., 2011). One non-species-specific autoinducer used as “universal language,” autoinducer 2 (AI-2) (Figure 2C), is used for intra- and inter-species communication regardless of whether they are gram-negative or gram-positive bacteria (Lowery et al., 2008). Moreover, autoinducer 3 (AI-3) produced by intestinal bacterial species, such as enterohemorrhagic *Escherichia coli* (EHEC) O157:H7, is involved in pathogen-host interactions (Sperandio et al., 2003; Walters et al., 2006). In addition to these common QS signaling molecules, communication by CAI-1, namely (S)-3-hydroxytridecan-4-one (Figure 2D), is used by *V. cholera* (Higgins et al., 2007). Relatively simple fatty acid derivatives called diffusible signal factors (DSFs) are used by *Burkholderia cepacia* and *Helicobacter pylori*, respectively (Deng et al., 2010; Tanaka et al., 2011). Diffusible extracellular factor (DF) is used by *Xanthomonas campestris* (He et al., 2011). Pseudomonas quinolone signaling (PQS) is identified in *P. aeruginosa* (Bala et al., 2013, 2014). Dialkylresorcinol is used by *Photorhabdus asymbiotica* (Brameyer et al., 2015).

AHLs are the most common class of autoinducers and are present in approximately 10% of proteobacteria isolated from various ecological niches (Tan et al., 2015). AHL signaling molecules possess a homoserine lactone (HSL) moiety, acyl side chains with 4–18 carbons that determine the structure diversity and a substitution primarily belonging to three types: (a) simple acyl, (b) 3-hydroxyacyl, and (c) 3-oxoacyl groups (Marketon et al., 2002; Schaefer et al., 2008; Thiel et al., 2009; Galloway et al., 2011; Savka et al., 2011). The biological activity of these molecules depends on the stereochemistry (Geske et al., 2008; Galloway et al., 2011). In the LuxI/R system, LuxI proteins are responsible for the synthesis of AHLs, and LuxR proteins combine with AHLs to control target gene expression. This system is the most prevalent QS regulatory system in gram-negative bacteria (Miller and Bassler, 2001). However, LuxR orphans or solos, i.e., LuxR homologs without the cognate autoinducer synthase LuxI, were also reported, such as SdiA in *Salmonella enterica* and *E. coli* (Ahmer et al., 1998; Kanamaru et al., 2000), QscR in *P. aeruginosa* (Fuqua, 2006) and PluR in *Photorhabdus luminescens* (Brameyer et al., 2015). These LuxR orphans contain an AHL-binding domain at the N-terminus to bind to endogenous AHLs (Lequette et al., 2006), exogenous AHLs (Yao et al., 2006) or other novel signals (Brameyer et al., 2015) as well as a DNA-binding helix-turn-helix (HTH) domain at the C-terminus.

AI-2, which represents a universal “language” to facilitate interspecies communication, is a byproduct of the detoxification of S-adenosylmethionine (SAM), which is catalyzed by S-ribosylhomocysteine lyase (LuxS, EC 4.4.1.21) (Xavier and Bassler, 2005a; Lowery et al., 2008). Studies of the recognition and signal transduction of AI-2 have focused primarily on the LuxP-based system of *V. harveyi* (Neiditch et al., 2005; Defoirdt et al., 2008) and LasB-based systems of *E. coli* (Xavier and Bassler, 2005b) and *Salmonella* sp. (Taga et al., 2003; Zhu et al., 2008). Different bacteria responded to AI-2 in different manners, because differentiation of AI-2 related signaling primarily occurred at the level of transduction. However, recent studies demonstrated that the AI-2 of *Campylobacter jejuni* strain NCTC 11168e (Holmes et al., 2009) and LuxS in *Vibrio ichthyoenteri* (Li et al., 2010) may not be involved in cell-cell communication or the autoinducer-regulated phenotypes of cell growth, biofilm formation, or virulence gene expression.

Because prokaryotes and eukaryotes have coexisted and coevolved for millions of years, the existence of interkingdom communication inevitably evolved (Shiner et al., 2005; Lowery et al., 2008; González and Venturi, 2013). The “languages” of AHLs (Joint et al., 2002; Bortolotti et al., 2015), epinephrine/norepinephrine (Sperandio et al., 2003) and AI-3 systems (Clarke et al., 2006) are used as signaling molecules in interkingdom communication. The first example of QS signaling molecules modulating the host cells was N-(3-oxododecanoyl)-L-homoserine lactone (3-oxo-C12-HSL) secreted by *P. aeruginosa* (Telford et al., 1998). The immunomodulatory activity of 3-oxo-C12-HSL was further shown to inhibit T-cell differentiation and cytokine production by a mechanism involving an early step in T-cell activation (Ritchie et al., 2005), but this signaling molecule induced the expression of HLA-G, the non-classical class I human leukocyte antigen (HLA), in monocytes and T-cells (Bortolotti et al., 2015). The eukaryotic response to QS is also interesting, and the representative example is the interference of bacterial QS with furanone produced by the red alga *Delisea pulchra* (Givskov et al., 1996). Since then, the signaling interfering activity of brominated furanones has been extensively explored.

## Inhibition and activation of QS systems in gram-negative bacteria

### Inhibition of QS systems

**Figure 3:**
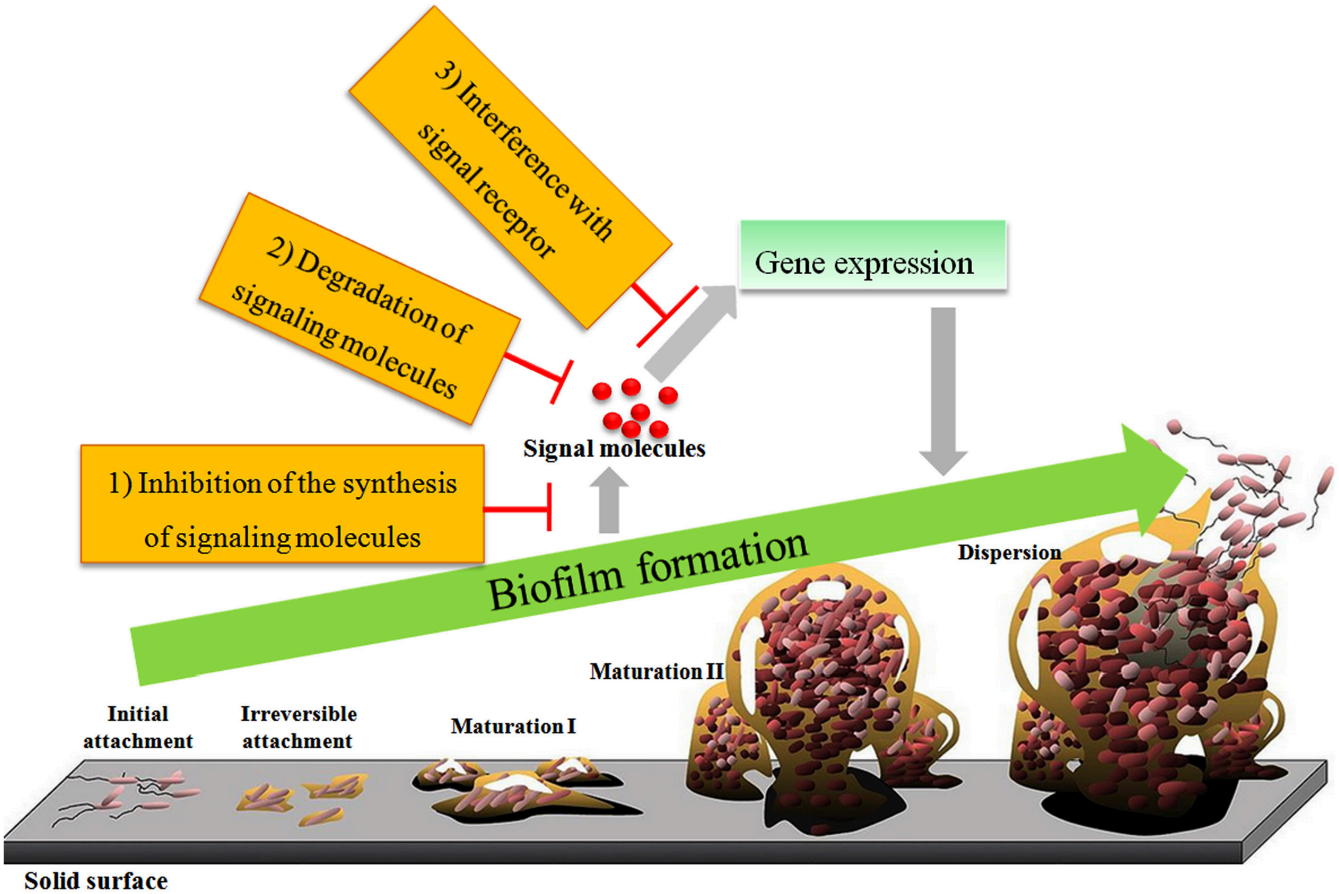
**Schematic diagrams of biofilm formation and the interruption of QS systems**.

Currently, interference with the QS systems is primarily achieved by inhibiting the synthesis of autoinducers, degrading autoinducers, interfering with autoinducer receptors, or inhibiting the autoinducer/receptor complex formation (Figure 3; Lade et al., 2014). The molecules responsible for inhibition of autoinducer-induced QS systems or the autoinducer-regulated phenotype are called quorum-sensing inhibitors (QSIs). QSIs include furanones and their related structural analogs (Figure 2E; Zang et al., 2009; Liu et al., 2010; Steenackers et al., 2010), bismuth porphyrin complexes (Galkin et al., 2015), glycosylation reagents of glycosylated flavonoids (Figure 2F; Brango-Vanegas et al., 2015), and glycomonoterpenols (Mukherji and Prabhune, 2015), heavy metals (Vega et al., 2014) and nanomaterials (Wagh Nee Jagtap et al., 2013; Miller et al., 2015; Singh et al., 2015). The inhibitory effect of furanones is primarily due to their structural similarity to AHLs, but some case studies also showed that furanones may function through degrading the LuxR-type protein (Manefield et al., 2002) or decreasing the DNA-binding activity of the transcriptional regulator protein LuxR (Defoirdt et al., 2007b). In addition to AHL autoinducers, furanones also disrupt the AI-2 biosynthetic pathway by covalently modifying and inactivating LuxS (Zang et al., 2009). In addition to furanones, allyl benzo[b]thiophene-3-carboxylate 1,1-dioxide (Figure 2G) targeted LuxPQ (Zhu et al., 2012), thiazolidinediones and dioxazaborocane targeted LuxR in *V. harveyi* (Brackman et al., 2013) were also reported to be AI-2 inhibitors. Alkylamine-modified cyclodextrins (Morohoshi et al., 2013) and homoserine lactone (HSL) aptamer (Figure 2H; Zhao et al., 2013) inhibited the QS system through the formation of spatial conformations, whereas silicon dioxide nanoparticles functionalized with β-cyclodextrin reduced the luminous output of *V. fischeri* through binding of AHLs to the nanoparticles and removal of the AHLs from the immediate bacterial environment (Miller et al., 2015). Recently, ureidothiophene-2-carboxylic acids were described as inhibitors of the PQS-QS enzyme PqsD in *P. aeruginosa* (Sahner et al., 2015).

**Figure 4:**
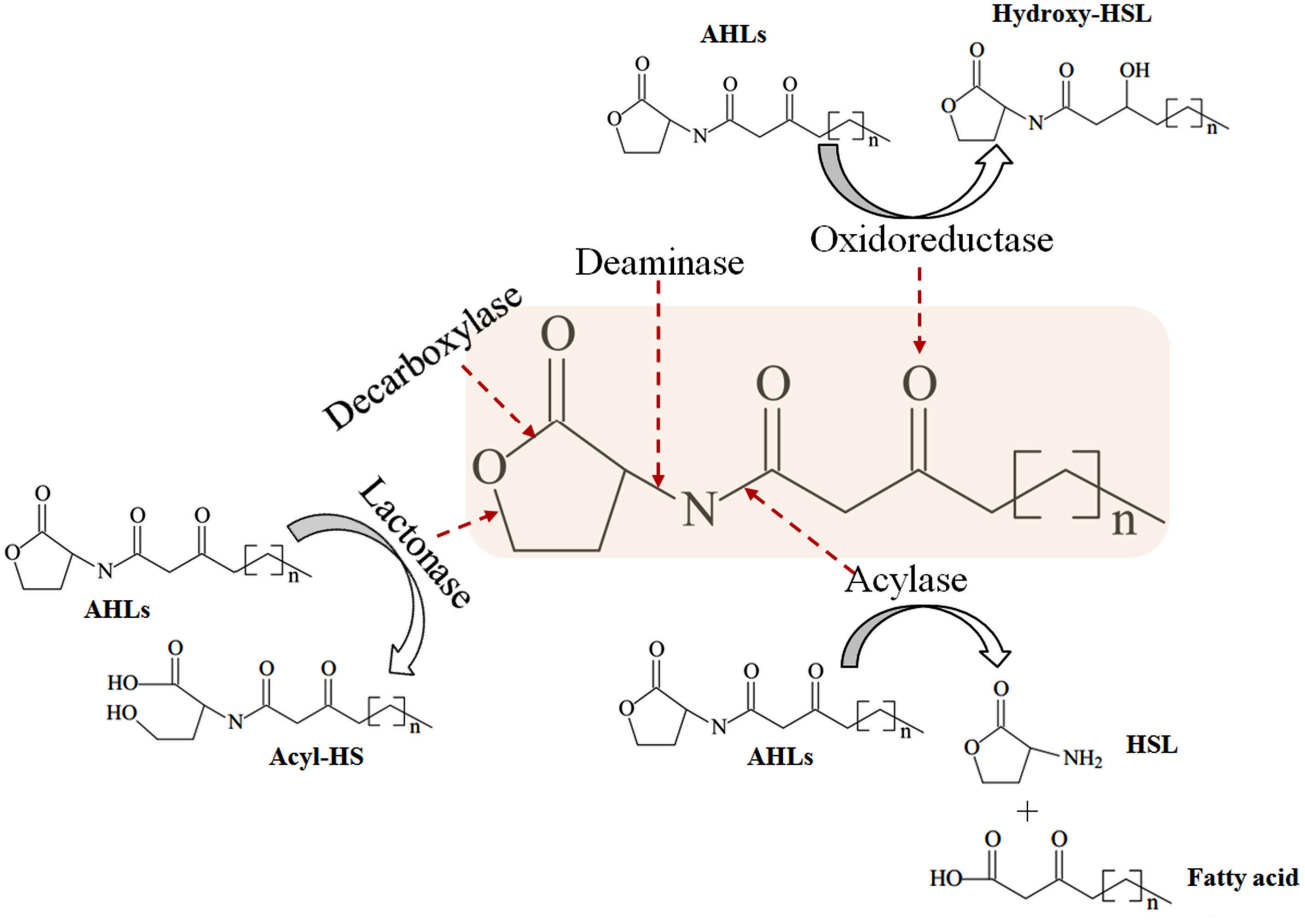
**Modes of AHL-degrading enzymes and their catalytic sites**. Revised from Chen et al. (2013).

In addition to small molecule QSIs, enzymatic degradation is an alternative approach to inhibit bacterial signaling molecules. AHL-lactonases and decarboxylases hydrolyze lactone rings, whereas AHL-acylases and deaminases cleave the acyl side chain (Figure 4; Amara et al., 2011). Acylases that hydrolyze the amide moieties of AHLs have been identified in *Streptomyces* sp. (Park et al., 2005), *P. aeruginosa* (Sio et al., 2006), *Comamonas* (Uroz et al., 2007), *Pseudomonas syringae* (Shepherd and Lindow, 2009), *Streptomyces* sp. (Ueda et al., 2010), *Bacillus cereus* (Sunder et al., 2012), and *Kluyvera citrophila* (Mukherji et al., 2014). Metallo-beta-lactamases hydrolyze the core lactone ring of AHL signaling molecules and are noted in *Acinetobacter* sp. (Kang et al., 2004), *Rhodococcus* sp. (Park et al., 2006; Uroz et al., 2008), *Agrobacterium tumefaciens* (Liu et al., 2007) and *Chryseobacterium* sp. (Wang et al., 2012). Oxidoreductases from *Rhodococcus erythropolis* (Uroz et al., 2005) and *Bacillus megaterium* (Chowdhary et al., 2007) target the keto group of 3-oxo-HSLs or the acyl side chain itself. Immobilized enzymes on solid materials, such as magnetic enzyme carriers (MECs) (Yeon et al., 2009b; Lee et al., 2014), nanofiltration (NF) (Kim et al., 2011), microporous hollow fiber membranes (Jahangir et al., 2012; Oh et al., 2012), and encapsulation of QQ bacteria *Rhodococcus* sp. (Oh et al., 2012; Kim et al., 2013), have been used to inhibit QS signaling systems.

### Activation of QS systems

Compared with the well-studied QSIs, research on activation of QS is in its infancy. Agonistic analogs of QS signaling molecules were believed to trigger the activation of the host defense system and thus allow resistance to develop via activation of virulence factors at low population densities (Defoirdt et al., 2004). N-(3-nitro-phenylacetanoyl)-L-homoserine lactone (Figure 2I) was one of the first reported super-agonists of AHLs to trigger bioluminescence in *V. fischeri*, increasing activity relative to native AHLs (Geske et al., 2007a,b). In the non-AHL-producing *Salmonella enterica* serovar Typhimurium, the LuxR homolog SdiA was activated by N-(3-oxo-acyl)-homocysteine thiolactones (3O-AHTLs, Figure 2J) and N-(3-oxo-acyl)-trans-2-aminocyclohexanols (Figure 2K) at concentrations lower than those of most active AHLs (Janssens et al., 2007). Naphthyl-DPD (Figure 2L), the agonistic analog of AI-2 precursor DPD, is an efficient synergistic agonist in *V. harveyi* even at low nanomolar concentrations. The synergistic effect is based on binding of one DPD unit to one LuxPQ domain and binding of naphthyl-DPD to the second LuxPQ domain, allowing a favorable conformational change that leads to early induction of QS activity (Mandabi et al., 2015). Chemicals such as (4S,5R)-dihydroxyhexanediones (DHDs, Figure 2M) is not only a synergistic agonist for the LsrB receptor in *E. coli* but also an agonist in *V. harveyi* with a LuxP receptor, displaying an EC_50_ = 0.65 μM. This activity is the strongest agonistic activity reported thus far in *V. harveyi* (Rui et al., 2012).

### Regulation of QS systems by sub-lethal antibiotics

Interestingly, sub-lethal antibiotics also function as QS system mediators. Azithromycin, ceftazidime, and ciprofloxacin (CPR) reduce the production of the AHLs, most likely due to the changes in membrane permeability in the presence of antibiotics that decreased flux of AHLs and expression of QS-regulated genes (Skindersoe et al., 2008). Similarly, streptomycin at sub-lethal concentrations reduced 3-OH-C12-HSL levels through the down-regulation of *abaI* and *A1S_0112* from *Acinetobacter baumannii* (Niu et al., 2008; Clemmer et al., 2011; Saroj and Rather, 2013). Aspirin at sub-lethal concentrations is an efficient inhibitor of QS, virulence, and toxins in *P. aeruginosa* via the interaction between the aspirin aryl group and Tyr-88 of the LasR receptor by strong π–π stacking interactions (El-Mowafy et al., 2014). However, some antibiotics, such as CPR, metronidazole, and tinidazole, exhibited a dose-dependent augmentation in response to QS systems, exhibiting an AHL-like effect. Alternatively, at the concentrations tested, these antibiotics may themselves act as QS signaling molecules (Struss et al., 2012). The contradictory effect of CPR on QS systems may be due to the different reporter cells used, e.g., *P. aeruginosa* PAO1 (Skindersoe et al., 2008) and *E. coli* expressing *P. aeruginosa* QS systems (Struss et al., 2012).

## Quorum sensing and environmentally related processes

### Quorum sensing and wastewater treatment systems

In wastewater treatment systems, one critical step involves removal of organic pollutants by biological treatments based on the microbial decomposition of pollutants into small molecules with less or even no toxicity, which simultaneously generates energy for microbial metabolism and the building blocks for cell synthesis. This method is now considered an essential step in wastewater treatment system and has been classified into two primary groups: fixed-film systems and activated sludge systems (Feng et al., 2013). QS systems are involved in the following aspects of these processes.

### Involvement in biofilm formation

Biofilms consist of bacterial cells surrounded by an extracellular matrix consisting of secreted proteins, polysaccharides, nucleic acids, and dead cells; biofilm development progresses through the stages of initial attachment, irreversible attachment, maturation I, and maturation II (Figure 3; Parsek and Greenberg, 2005). AHLs freely diffuse in the microenvironment and are involved in biofilm formation in *P. aeruginosa* (Davies et al., 1998), *Pantoea stewartii* (Koutsoudis et al., 2006), *Acinetobacter* sp. (Kang and Park, 2010), and *Serratia plymuthica* (Liu et al., 2011). In *P. aeruginosa*, the *las* QS system regulates biofilm formation through control of the *pel* operon that encodes the glucose-rich matrix exopolysaccharide, a component of the biofilm matrix (Davies et al., 1998; Sakuragi and Kolter, 2007). However, exogenous 3-oxo-octanoyl-L-homoserine lactone (C8-oxo-HSL) increased the growth rate of *P. aeruginosa* cells on an ultra-filtration membrane biofilm without influencing the production of extracellular polymeric substances (EPS, Xia et al., 2012). The correlation between QS and EPS production in a growing biofilm under various conditions was modeled, illustrating the benefits of QS regulation by developing a thick, protective layer of EPS or by clogging the environment with biomass to secure a nutrient supply and outcompete other colonies (Frederick et al., 2011).

**Figure 5:**
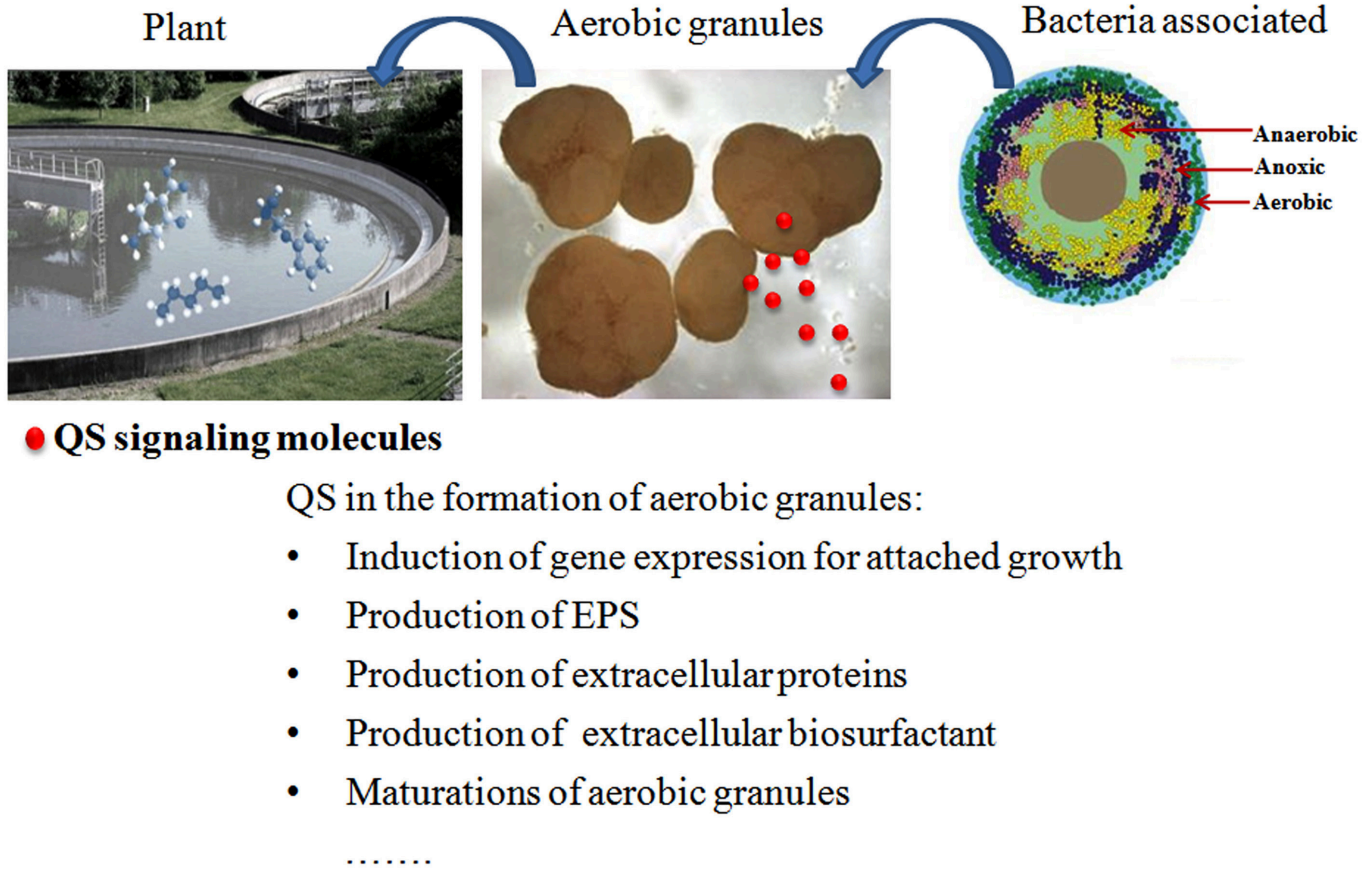
**Schematic diagrams of aerobic granulation, attached bacteria, and function of QS signaling molecules**.

In the natural environment, biofilm formation by microorganisms can be beneficial or detrimental from the human perspective. Biofilms formed by degrading microorganisms are generally used to remove hydrocarbons, heavy metals, and nutrients to improve the mineralization process, as they offer a sessile, protective environment with optimal pH, solute concentrations and redox potential (Singh et al., 2006). Aerobic granules cultivated in sequencing batch reactors (SBRs) for biological wastewater treatment are considered a special type of biofilm and share many features of biofilm systems (Liu and Tay, 2002; Yang et al., 2004). Aerobic granules possess the merits of high stability and flexibility, low energy requirements, reduced footprint, aerobic, and anoxic zones inside the granules, and reduced investment and operational costs in practical applications compared with conventional activated sludge (Aqeel et al., 2015; Long et al., 2015; Pronk et al., 2015; Wagner et al., 2015). In aerobic granules, a gradient microbial population of aerobic bacteria, anaerobic bacteria, and dead microbial cells under the granule surface co-exists due to the presence of oxygen and nutrient gradients (Figure 5; Liu et al., 2002). AHLs produced by some of these bacteria, e.g., *Aeromonas* sp., *Pseudomonas* sp. and *Acinetobacter* sp., were detected (Valle et al., 2004; Morgan-Sagastume et al., 2005; Chong et al., 2012; Yong and Zhong, 2013b). Both the autoinducers of AHL and AI-2 were observed in the biomass during the period of aerobic granulation, and this phenomenon was frequently ATP-dependent (Xiong and Liu, 2010; Zhang et al., 2011; Jiang and Liu, 2013; Wang et al., 2014). AHLs at picomolar to nanomolar concentrations are strongly and mostly positively correlated with the initiation of granulation, formation of highly structured granules, and maintenance of granular structure in the granulation ecosystem.

Mature granules or their extracts induced the bacterial attached-growth state with a high adhesion capability, contributing to the initial cell attachment and subsequent biofilm growth on a Flow-Cell cover slide, thus accelerating the formation of aerobic granules from conventional activated sludge (Ren et al., 2010, 2013). This effect may be attributed to the increased activity of AHL signaling molecules contained in the mature granules or their extracts (Li et al., 2014). The correlation between QS signaling molecules and the production of EPS, a significant factor promoting the maintenance of aerobic granular structures during the development of aerobic granules and biofilm growth, was confirmed (Jiang and Liu, 2012; Li et al., 2014; Tan et al., 2014). AHLs also regulated the production of extracellular biosurfactants (or act as biosurfactants) (Daniels et al., 2006) and extracellular proteins (Lv et al., 2014a). The involvement of QS in the formation of aerobic granules was also confirmed using quorum quenching (QQ). A high rate of QQ activity was presented in the floccular sludge. However, when the floccular biomass was transformed into granular sludge, the QQ activity of the community was reduced by 30%, whereas the levels of C4–C8 AHLs associated with the initiation of granulation increased up to 10- to 100-fold. However, when the granule disintegrates, C6-HSL, and C8-HSL levels decreased with the reduced sludge particle size (Tan et al., 2014, 2015).

Biofouling is an adverse effect of the biofilm that forms by the attachment of microorganisms on the surface of various solid matrixes. This condition is the primary hindrance during the development and application of membrane bioreactors (MBR) and consumes 60% of MBR operating fees (Drews, 2010). AHL autoinducers from C4-HSL to C14-HSL (Kim et al., 2009, 2013; Yeon et al., 2009a; Cuadrado-Silva et al., 2013) as well as AI-2 (Davies et al., 1998; Xu and Liu, 2010) have frequently been detected during biofouling in the MBR environment. Furthermore, a positive correlation between AHLs and membrane biofouling was confirmed because the strong AHL activity corresponded to an abrupt increase in the transmembrane pressure (TMP) in a fouled MBR (Yeon et al., 2009a). The presence of autoinducers in the biofilm and the relationship between autoinducers and biofilm formation phenotype, TMP and EPS measurements suggest alternative strategies for controlling membrane biofouling via interference of the autoinducer-mediated QS systems. In fact, the activation and inhibition of QS systems are two antagonistic coexisting processes among various bacterial communities in different wastewater treatment systems (Song et al., 2014). Chemicals, such as 5-hydroxy- 3[(1R)-1-hydroxypropyl]-4-methylfuran-2(5H)-one, (5R)-3,4-dihydroxy-5-[(1S)- 1,2-dihydroxyethyl]furan-2(5H)-one, kojic acid (Dobretsov et al., 2007, 2011), and non-halogenated 2(5H)-furanone (Ponnusamy et al., 2010), are QSIs that control the formation of biofilms. Given the toxicity of furanones, non-toxic agents were preferred alternative strategies to control biofouling, which may mitigate membrane biofouling without disturbing bacterial growth. Vanillin was reported to reduce the production of AHL autoinducers and decrease the biofilm formation of *Aeromonas hydrophila* on a membrane at very low concentrations (Ponnusamy et al., 2009, 2013). Further application of vanillin to a reverse osmosis membrane in a CDC reactor exhibited 97% biofilm reduction (Kappachery et al., 2010). D-tyrosine significantly reduced the microbial attachment on a solid matrix through its inhibitory effect on the synthesis of AI-2, eDNA and extracellular polysaccharides and proteins (Xu and Liu, 2011). In addition to small chemicals, acylases are also used to reduce the biofouling from fouled reverse osmosis membranes (Paul et al., 2009; Yeon et al., 2009a; Kim et al., 2012). To overcome the limitations of a short half-life and low efficiency using free enzymes, immobilized acylase bound to magnetic particles was used to inactivate the AHL autoinducers to reduce biofilm formation and enhance membrane permeability (Yeon et al., 2009b). Alternatively, more feasible and economic methods based on the encapsulation of *Rhodococcus* sp. BH4 and recombinant *E. coli*-producing AHL-lactonase in microporous membranes were developed. The biofilm formed on the membrane in the microbial vessel reactor was approximately half of the total attached biomass and thinner and sparser compared with that in the control reactor (Oh et al., 2012). Lactonase from *Rhodococcus* sp. BH4 is responsible for the degradation of a wide range of AHLs and thus inhibition of biofouling (Oh et al., 2013). To overcome the limitation of transference of mass and AHLs from the mixed liquor to the microbial vessel encapsulated *Rhodococcus* sp. BH4, cell entrapping beads (CEB), i.e., moving beads of alginate containing *Rhodococcus* sp. BH4, were developed with a better capacity to mitigate biofouling. In MBRs, the time to achieve a TMP of 70 kPa in the presence of CEBs was 10-fold longer than that in the absence of CEBs (Kim et al., 2013). Another macrocapsule consisting of the membrane coating layer and alginate core with *Rhodococcus* sp. BH4 was developed, and it decreased to half the total amount of biocakes (Kim et al., 2015). *Pseudomonas* sp. 1A1 probably producing AHL-acylase to degrade AHLs were also encapsulated in the ceramic microbial vessel, leading to significantly reduced polysaccharides and proteins (Cheong et al., 2013, 2014). However, although disruption of QS systems has been employed to mitigate biofouling, the above findings have yet to be used in practice.

### Optimization of the composition of the bacterial community

It is generally accepted that removal of different pollutants by microorganisms is dominated by the percentage of and interaction between different microbes. In activated sludge communities from industrial wastewater treatment systems, a dominant functional member of the *Thauera* genus was transiently supplanted by a member of the *Comamonas* genus in response to the exogenous AHL. In addition, phenol degradation rates were restored to the original higher levels (Valle et al., 2004). In another long-term operated bioreactor, greater than 50% of the top 50 most abundant community members exhibited a strong positive correlation with at least one AHL and granulation (Tan et al., 2014). *Xanthomonadaceae* (Tan et al., 2014) and *Flavobacterium* (Lv et al., 2014b) in aerobic granules correlated with increased AHLs content. In contrast, an AHL signal degrader from the *Comamonadaceae* family exhibited a negative correlation with both the AHL concentration and granulation (Tan et al., 2014).

### Strengthen the biodegradation/biotransformation pathways in gram-negative bacteria

QS bacteria can degrade or transform phenol (Valle et al., 2004; Yong and Zhong, 2010), hexadecane (Kang and Park, 2010), phenanthrene, or pyrene (Huang et al., 2013), nitrogen (Toyofuku et al., 2007, 2008; De Clippeleir et al., 2011) and total organic carbon (Zhang et al., 2015). The first evidence demonstrating AHL involvement in biodegradation was observed in phenol degradation by activated sludge. The addition of 2 μM AHLs could maintain the phenol biodegradation ability for a longer period. However, this work did not present any evidence for the QS effect on bacterial biodegradation or pollutant metabolism (Valle et al., 2004). A more detailed study examined the RhlI/R QS system in a *P. aeruginosa* organic pollutant degrader from industrial and municipal wastewater and its involvement in biodegradation and denitrification. Mutants of △*rhlI* and △*rhlR* exhibited increased denitrification activity (Toyofuku et al., 2007) and reduced phenol degradation (Yong and Zhong, 2010, 2013b), and the exogenous addition of the cognate AHL molecules or AHL extracts induced the QS mutant strains to exhibit denitrification activity similar to the wild-type strain. The repression of mutant Δ*rhlI* but not mutant Δ*rhlIR* could also be relieved by supplementation with AHL extracts or synthetic C4-HSL. These data supplied concrete evidence to demonstrate the involvement of the QS system in biodegradation (Yong and Zhong, 2010, 2013b). *abaI* encodes the autoinducer synthases of the LuxI family in another important aromatic compound degrader, *Acinetobacter* sp. (Smith et al., 2007; Niu et al., 2008; Anbazhagan et al., 2012), which is also involved in biofilm formation and hexadecane biodegradation. Its activity is enhanced by adding exogenous 3-OH-C12-HSL (Kang and Park, 2010). However, in another case study in activated sludge, short chain AHLs were responsible for nicotine degradation and had an increased effect on *Acinetobacter* sp. TW colonization, whereas long chain AHLs are secreted and contribute to resistance to the toxin nicotine (Montgomery et al., 2013; Wang et al., 2013a,b).

The expression and activity of enzymes involved in biodegradation are regulated by autoinducers and QS systems. During the degradation of anthranilate and phenol by *P. aeruginosa*, expression of catechol-1,2-dioxygenase (C12O) and catechol 2,3-dioxygenase (C23O) was significantly enhanced by the addition of N-decanoyl-L-homoserine lactone (C10-HSL), N-octanoyl-L-homoserine lactone (C8-HSL) or C4-HSL (Chugani and Greenberg, 2010) through the rhlI/R QS system (Yong and Zhong, 2013b), respectively. In denitrification, the rhlI/R QS system repressed the promoter activities of *nark1, nirS, norC*, and *nosR* and thus the corresponding activities of ${\text{NO}}_{3}^{-}$ reductase, ${\text{NO}}_{2}^{-}$ reductase, and NO reductase were higher in the mutant of △*rhlR* (Toyofuku et al., 2007). Moreover, the reduction of NO reductase and ${\text{NO}}_{3}^{-}$ reductase and induction of ${\text{NO}}_{2}^{-}$ reductase at the post-transcriptional level are also regulated by the PQS-based QS system in *P. aeruginosa* (Toyofuku et al., 2008).

### Quorum sensing and aquaculture

QS systems were extensively confirmed to be involved in regulation of virulence of pathogens in aquaculture. Virulence factors of the opportunistic pathogen *V. harveyi*, including extracellular toxin (Manefield et al., 2000), siderophore (Lilley and Bassler, 2000), metalloprotease (Mok et al., 2003), type III secretion system (Henke and Bassler, 2004), phospholipase, caseinase, and gelatinase (Natrah et al., 2011b), are regulated by QS systems. In a number of other *Vibrio* species, such as *V. cholerae, V. vulnificus, V. anguillarum, V. splendidus, V. aestuarianus*, and *V. vulnificus*, the expression of metalloproteases is also modulated by QS (Decker et al., 2013; Ha et al., 2014). More direct evidence revealed that attenuation of QS in pathogenic *Aeromonas* spp. and *V. campbellii* resulted in significantly reduced mortality toward their respective hosts burbot (Natrah et al., 2012), larvae of brine shrimp and giant freshwater prawn (Pande et al., 2013).

Due to the close association between the QS system and virulence of aquatic pathogens, ecological strategies are the preferred option to overcome the problems of acquisition of antibiotic resistance and the spread of resistant genes when antibiotics or disinfectants are used to treat bacterial diseases (Homem and Santos, 2011; Singh, 2015). QSIs, such as the natural furanones of (5Z)-4-bromo-5-(bromomethykene)-3-butyl-2(5H)-furanone (Figure 2E; Defoirdt et al., 2006, 2007a,b), halogenated furanones (Zang et al., 2009), furanones F2 (Liu et al., 2010), brominated 3-alkyl-5-methylene-2(5H)-furanones, and alkylmaleic anhydrides (Steenackers et al., 2010), and other chemicals of thiophenone (Z)-4-((5-(bromomethylene)-2-oxo-2,5-dihydrothiophen-3-yl)-4-oxobutanoic acid) (Figure 2N; Defoirdt et al., 2012; Pande et al., 2013), are effective in attenuating bacterial virulence. Because the therapeutic index of furanones is likely too low to be used in the aquaculture given their relatively high toxicity to higher organisms (Defoirdt et al., 2006; Pande et al., 2013), non-toxic natural compounds were explored. Cinnamaldehyde (Figure 2O), a QS-disrupting compound, protects burbot (*Lota lota* L.) larvae from *A. hydrophila* and *A. salmonicida* (Natrah et al., 2012), the giant freshwater prawn *Macrobrachium rosenbergii* from *V. harveyi* (Pande et al., 2013) and brine shrimp larvae from *V. harveyi* (Brackman et al., 2013). Coumarin (Figure 2P) was effective in reducing the biofilm formation of *E. tarda, V. anguillarum, E. coli*, and *Staphylococcus aureus*. The above four strains use three types of autoinducers, e.g., AHLs, AI-2, and *agr*, suggesting that coumarin could be used as a universal QS inhibitor to attenuate bacterial disease in aquaculture (Gutiérrez-Barranquero et al., 2015). The trend of using probiotic microorganisms to control disease in aquaculture is encouraging, as they can disrupt the QS systems of pathogens. The positive effect of AHL-degrading *Bacillus* sp. may result from the degradation of autoinducers in addition to the production of growth-inhibiting substances (Defoirdt et al., 2004). The microalgae *Chlorella saccharophila* CCAP211/48, which is commonly used in aquaculture, exhibited stable inhibitory activity on *V. harveyi* with the production of QS antagonistic metabolites that have not been reported previously in microalgae (Natrah et al., 2011a).

## Opportunities and challenges

QS systems dynamically control gene expression in a cell density-dependent manner, based on the production, secretion, and detection of autoinducers. The concentration of autoinducers increases with the cell density. Once a threshold level is achieved, autoinducers induce the expression of QS-dependent target genes to facilitate environmental adaptation. As a global gene regulatory network, autoinducer-induced QS systems are involved in the regulation of bacterial virulence, conjugative plasmid transfer, sporulation, biofilm formation, antimicrobial peptide synthesis, and symbiosis. Future studies and applications of autoinducer-induced QS systems may focus on the following aspects.

- Although increasing attention has been paid to the ecological applications of QS regulation of biofilms, the mechanism by which bacterial autoinducer-induced QS systems operate in the “city” of biofilms remains rudimentary. A clear understanding is required to explore how the biofilm influences the initial QS system and subsequently the QS-regulated gene expression profiles and the development of multi-species biofilms.
- The QS system plays an important role in biodegradation. Thus, QS manipulation may become increasingly important in biodegradation and environmental bioremediation at the population/community and molecular level. However, QS systems have not gained the attention that they deserve in biodegradation compared with their function in many other biological processes. Further, study of the QS systems in biodegradation may accelerate the speed and rate of pollutant degradation. Additionally, the improvement of biodegradation efficiency by engineering the QS system is in progress and is a strategy that deserves more attention in the future. Moreover, the aromatic degradation pathway is a source of new signaling molecules and/or QQ signaling molecules. Thus, QS regulation of aromatic degradation processes and the latter as sources of signaling molecules are also notable topics.
- The design of novel antimicrobial agents based on QS regulation to control bacterial infections is recommended, despite that it will likely be many years before clinically safe and effective QSIs are available for real-life applications. Considering the fact that microbial consortia are composed of different species and a wide range of QS-regulated gene expression, it should be noted that the entire QS-regulated network would not be affected by the QSI of a particular QS pathway.

Developments in the field of QS and the QS-regulated behaviors in the natural environment and host will clearly address these questions. The answers to these open questions will undoubtedly bring new insights and surprises.

## Funding

This work was financially supported by the National Natural Science Foundation of China (NSFC) grant 31200041, the Zhejiang Provincial Natural Science Foundation of China (LR14C190001), the Program of Science and Technology of Ningbo city of China (2015C50057), the Young Academic Leaders in Colleges and Universities in Zhejiang Province (pd2013099), the Zhejiang Open Foundation of the Most Important Subjects (xkzsc1408), and the K. C. Wong Magna Fund in Ningbo University.

### Conflict of interest statement

The authors declare that the research was conducted in the absence of any commercial or financial relationships that could be construed as a potential conflict of interest.
